# Highly-efficient full-color holographic movie based on silicon nitride metasurface

**DOI:** 10.1515/nanoph-2023-0756

**Published:** 2024-01-22

**Authors:** Masakazu Yamaguchi, Hiroki Saito, Satoshi Ikezawa, Kentaro Iwami

**Affiliations:** Department of Bio-Functions and Systems Science, Tokyo University of Agriculture and Technology, Koganei, Tokyo 184–8588 Japan; Department of Mechanical Systems Engineering, Tokyo University of Agriculture and Technology, Koganei, Tokyo 184–8588 Japan; Waseda Research Institute for Science and Engineering, Waseda University, Shinjuku, Tokyo 169–8555 Japan

**Keywords:** metasurface, holography, silicon nitride, dielectric, waveguide

## Abstract

Metasurface holograms offer various advantages, including wide viewing angle, small volume, and high resolution. However, full-color animation of high-resolution images has been a challenging issue. In this study, a full-color dielectric metasurface holographic movie with a resolution of 2322 × 2322 was achieved by spatiotemporally multiplexing 30 frames with blue, green, and red color channels at the wavelengths of 445 nm, 532 nm, and 633 nm at the maximum reconstruction speed of 55.9 frames per second. The high average transmittance and diffraction efficiency of 92.0 % and 72.7 %, respectively, in the visible range, were achieved by adopting polarization-independent silicon nitride waveguide meta-atoms, resulting in high color reproducibility. The superposition of three wavelengths was achieved by adjusting the resolutions and positions of target images for each wavelength while maintaining the meta-atom pitch constant. The improvement in diffraction efficiency was brought about by the optimization of etching conditions to form high-aspect vertical nanopillar structures.

## 1 Introduction

Holography is a technology for recording and reconstructing light wavefront and is promising as a three-dimensional display because it offers natural observation with the naked eye [1], [2]. Holograms are storage media that record interference between an object light and a reference light as a fringe pattern, which contains information on both the amplitude and phase of the former [3]. Conventional holograms have adopted photosensitive materials, for example, silver halide emulsions. A method has been proposed to record and playback movies by automatically feeding these materials frame by frame as a film roll [4]. However, this method cannot record images that do not exist in reality and can only record images under specific conditions, for example, a dark room.

Computer-generated holography (CGH) is a method for calculating the interference pattern based on diffraction theory and can reconstruct light propagating from virtual objects. The interference pattern is expressed as an amplitude or phase profile by diffractive optical elements (DOE) or spatial light modulators (SLM). Especially, SLM-based holograms offer dynamic properties, making it possible to realize movie reconstruction [5]. However, the SLM pixel size ranges from several to several dozen microns [6], resulting in a narrow viewing angle.

Metasurface holograms provide various advantages, including a wide viewing angle, small volume, and high resolution, because of the small pitch reaching the subwavelength scale [7], [8]. Metasurface is a planar branch of metamaterials and consists of microstructures (meta-atoms) arranged with a subwavelength pitch. As each meta-atom can control the amplitude, phase, and polarization of the incident light, a tailored wavefront can be achieved. This technology has been applied to various optical elements, including prisms [9], [10], lenses [11]–[18], polarizers [19], [20], and waveplates [21], [22]. By applying metasurfaces to holography, monochromatic stereoscopic images [23], color stereoscopic images [24], complex amplitude control [25], [26], and reconstruction of multiple image slices from a single metasurface with high efficiency [27], has been achieved have been realized. Taking advantage of the small sizes of metasurfaces, its application to AR/VR devices, for example, AR glasses [28], is expected. However, since the optical properties of metasurfaces are derived from their structure, it has been challenging to dynamically change the reconstructed image after fabrication.

Various efforts have been dedicated to realizing dynamic metasurface holograms [29], which can be broadly categorized into tunable metasurfaces and static multiplexing. The former includes pitch control with substrate expansion [30], [31], electro-optic modulation [32]–[34], chemical reaction [35]–[37], and phase change materials [38]–[40]. Most of these methods are limited to a binary, ternary, or quaternary display. Therefore, addressable approaches are used to partially show or hide spatial channels in order to increase the variety of information that can be presented. The latter utilizes polarization [41]–[43], incident angle [44], [45], topological charge [46]–[48], and in-plane space [49]–[52] to multiplex image frames. Although the first two are simple and have a high extinction ratio between the channels, their frame number is limited. Topological charge or optical angular momentum (OAM) multiplexing enables tens of frames because of the orthogonality of each OAM channel, resulting in a high playback speed with the help of an SLM. However, the resolution of the reproduced image tends to decrease as the number of frames increases, and the reconstructed images are limited to monochromatic primitive shapes expressed by point groups in the present.

Among the static multiplexing holograms, in-plane spatial multiplexing is most suitable for reconstructing movie consisting of complex image frames because it offers a suitable balance of playback speed, number of frames, and resolution for the observation by the naked eye. Gao et al. achieved a playback speed of 9523 frames per second (fps) with 28 frames at the wavelength of 633 nm using a digital micromirror device (DMD) [49]. Izumi et al. achieved a 48-frame movie with a speed of 30 fps at the wavelength of 633 nm using an automatic stage [50]. Yamada et al. realized a multicolor movie with 20 frames by superimposing three color channels with wavelengths of 445, 532, and 633 nm [51]. However, Yamada et al. used crystalline silicon with poor transmittance on the blue side, resulting in low light-utilization efficiency and color reproducibility problems, for example, the inability to produce white color. Thus, color holography that satisfies all of the following requirements: observable to the naked eye, high color reproducibility, high resolution, and sufficient playback speed, has not been realized.

In this study, a highly efficient full-color holographic movie was achieved with a maximum speed of 55.9 fps and a frame number of 30 by using a silicon nitride (SiN) dielectric waveguide meta-atom. The resolution was 2322 × 2322, and the reconstructed movie was able to be observed with the naked eye. SiN offers a high transmittance due to its low absorption coefficient at the whole visible region and low Fresnel reflection because the effective refractive index of the meta-atom becomes close to that of the substrate. Furthermore, the diffraction efficiency was improved up to 72.7 % by optimizing the fabrication condition to reduce the contribution of the zero-order light. As a result, a full-color holographic movie was achieved by spatiotemporally multiplexing 30 frames with blue, green, and red color channels at the wavelengths of 445 nm, 532 nm, and 633 nm.

## 2 Principles

Light propagation from a holographic plane located at *z* = 0 is calculated utilizing the angular spectrum method:

(1) $$U\left(k_{x},k_{y},0\right)=\overset{\infty }{\underset{-\infty }{\iint }}u\left(x,y,0\right)\exp\left\{-i\left(k_{x}x+k_{y}y\right)\right\}dxdy,$$

where *u*(*x*, *y*, 0) is the output waveplane of the hologram, and $U\left(k_{x},k_{y},0\right)$ is the angular spectrum. In this study, we utilize metasurface holograms composed of a grid of *M* × *N* pixels (meta-atoms) arranged in a square lattice with the pitch *p*. The coordinates (*x*, *y*) on the hologram is expressed using a pair of integers (*m*, *n*) as $x=\left(m-\frac{M-1}{2}\right)p,y=\left(n-\frac{N-1}{2}\right)p$ . Using this expression, the angular spectrum is discretized as:

(2) $$\begin{array}{ll} U_{\xi \eta }= & \overset{M-1}{\underset{m=0}{\sum }}\overset{N-1}{\underset{n=0}{\sum }}\exp\left(i\phi_{mn}\right)\\ & \;\times \exp\left\{-2\pi i\left(\frac{\left(m-\frac{M-1}{2}\right)\xi }{M}+\frac{\left(n-\frac{N-1}{2}\right)\eta }{N}\right)\right\}, \end{array}$$

where *ϕ*_*mn*_ is the discretized phase distribution on the hologram surface. (*ξ*, *η*) are indices for the discretized wave vectors of:

(3) $$\left(k_{x},k_{y}\right)=\frac{2\pi }{p}\left(\frac{\xi -M/2+1}{M},\frac{\eta -N/2+1}{N}\right).$$

From Equation (3), the maximum value of the wavenumber component for each axis is deduced as $k_{x\max}=k_{y\max}=\frac{\pi }{p}$ . A diffraction angle *θ* is given by:

(4) $$\theta =\sin^{-1}\left(\frac{\sqrt{k_{x}^{2}+k_{y}^{2}}}{2\pi /\lambda }\right).$$

Substituting Equations 3 into 4 yields:

(5) $$\theta =\sin^{-1}\left(\frac{\lambda }{p}\sqrt{{\left(\frac{\xi -M/2+1}{M}\right)}^{2}+{\left(\frac{\eta -N/2+1}{N}\right)}^{2}}\right).$$

**Figure 1:**
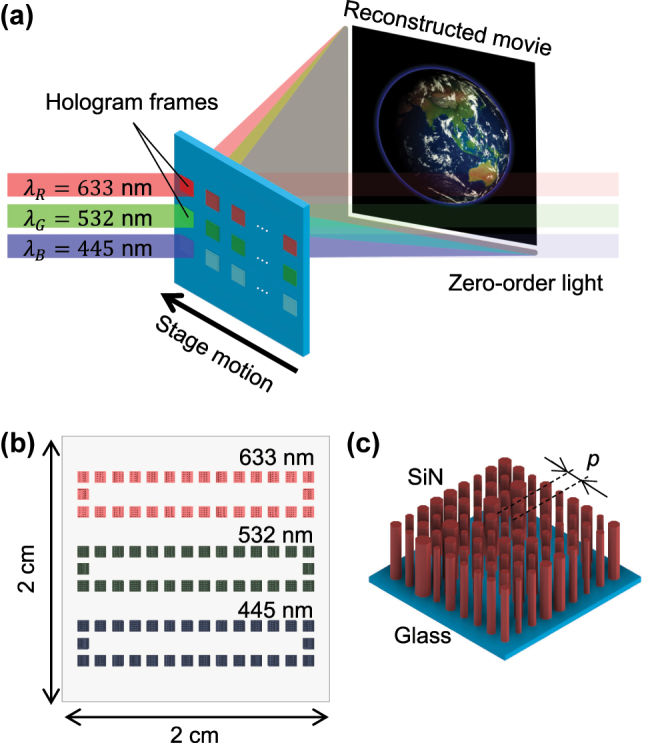
Schematic diagram of this study. (a) Schematic illustration of full-color metasurface holographic movie. Three-color lasers (wavelengths of 445 nm, 532 nm, and 633 nm) are irradiated on a frame on the specific channels, and an automatic stage sequentially scans the irradiation position to reconstruct the movie. (b) The layout of the metasurface holograms. For each wavelength, 30 frames of 0.79 × 0.79 mm^2^ hologram are arranged in a loopable pattern. (c) Structure of metasurface holograms. A square lattice of silicon nitride nanopillars with a regular octagonal cross-section is arranged on a glass substrate.

In this study, the full-color image is reconstructed by superimposing the three-wavelength channels, as shown in Figure 1. In order to successfully superimpose these color channels, it is necessary to make *θ* constant at each wavelength. From Equation (5), there are two ways to achieve this. The first is to keep the *λ*/*p* constant, and the second is to keep the product of *λ* and the root constant. Yamada et al. used the former method [51], which is complex because it requires multiple meta-atom sets. The latter method is simpler because it can use a single meta-atom set and is considered to be less susceptible to fabrication errors such as gap-dependent micro-loading effects. Therefore, it was adopted in this study because it is effective in terms of future scalability. The range occupied by the target image in the black-padded region is adjusted for each wavelength channel.

## 3 Design and fabrication of the full-color metasurface hologram with constant pitch

### 3.1 Design

The metasurface holograms fabricated in this study consist of SiN nanopillars with a regular octagonal cross-section arranged in a square lattice on a glass substrate (Figure 1(c)). The height of the nanopillar *h* and the pitch *p* are constant, and by changing the width of the nanopillar *w*, the effective refractive index *n*_eff_ is changed, producing different amounts of phase delay in the transmitted light. We adopted regular octagonal cross-sections to apply the character projection (CP) method for high-throughput electron beam lithography [53].

**Figure 2:**
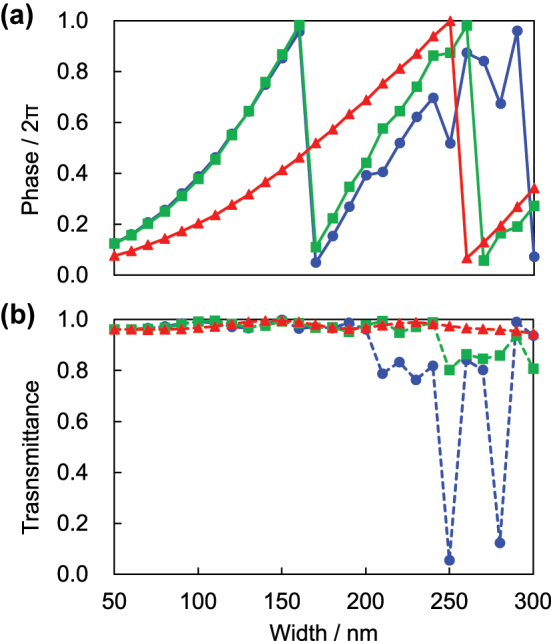
Calculated characteristics of meta-atoms. (a) Phase delay and (b) transmittance at each nanopillar width for incident wavelengths of 445 nm (blue), 532 nm (green), and 633 nm (red).

Electromagnetic field analysis of the nanopillars was performed using a commercial finite element method software, COMSOL Multiphysics 6.1 (COMSOL Inc., USA). The spectral refractive index *n* and extinction coefficient *κ* of SiN measured by an ellipsometer (M-2000DI-T, J. A. Woollam, USA) were used for the calculation. The incident light wavelengths were chosen as 445 nm (blue), 532 nm (green), and 633 nm (red). The height *h* and pitch *p* of the nanopillars were set to 1500 nm and 340 nm, respectively. The calculation results with varying pillar width from 50 to 300 nm are shown in Figure 2. Transmittance is decreased in the thicker region at 445 nm, which may be due to diffraction loss caused by the pitch not satisfying the non-diffraction condition on the substrate side *p* < *λ*/*n*_substrate_. However, high transmittance can be expected if a narrower width is used. Thus, based on these calculations, we adopted the width ranges of 90–190 nm for *λ* = 445 nm, 110–200 nm for *λ* = 532 nm, and 70–230 nm for *λ* = 633 nm.

Avoiding the zero-order light is an essential issue for holograms. There are methods to reduce the zero-order light intensity by improving diffraction efficiency or shifting the image through the off-axis approach [54]–[56]. Here, we adopt the black-padding method [51] for the simplicity.

**Figure 3:**
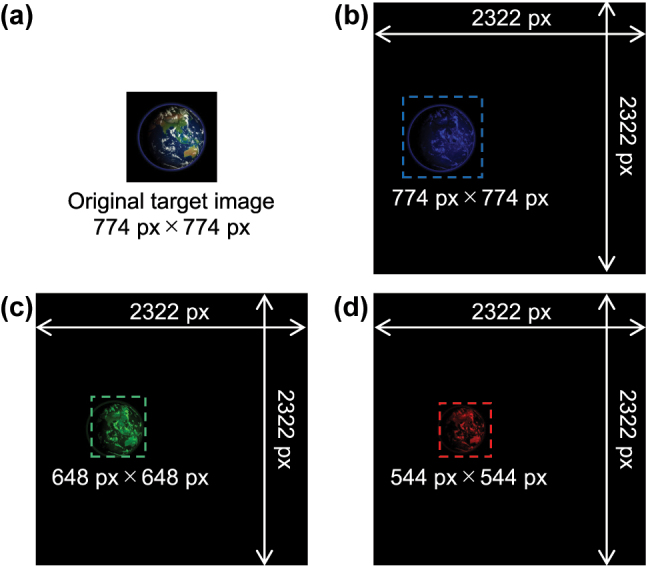
A selected frame of the target movie. (a) Original image and (b)–(d) its adjusted images for each color channel. The images are padded black to avoid overlap of zero-order light and conjugate image, and the range occupied by the target image in the black-padded region is adjusted for each wavelength channel to keep the diffraction angle constant.

Figure 3(a) shows a selected frame of the target movie with the resolution of 774 pixels × 774 pixels, which is cropped and resized from the original movie [57]. As discussed in equation (5), the resolution and position of the images in the black-padded region were adjusted to keep the diffraction angle *θ* constant for each wavelength channel as shown in Figure 3(b–d). The frame resolution of 2322 pixels × 2322 pixels, corresponding to the frame size of 0.79 mm × 0.79 mm with the 340-nm pitch, is determined to place 30 frames × 3 channels on a 20-mm-square substrate.

The phase profiles of the metasurface to reconstruct target frames were calculated using the iterative Fourier transform (IFT) method [58]. Using the Python library gdstk [59], the design layouts of the holograms were prepared by mapping the meta-atom set obtained in Figure 2 onto the calculated phase profiles.

### 3.2 Fabrication

**Figure 4:**
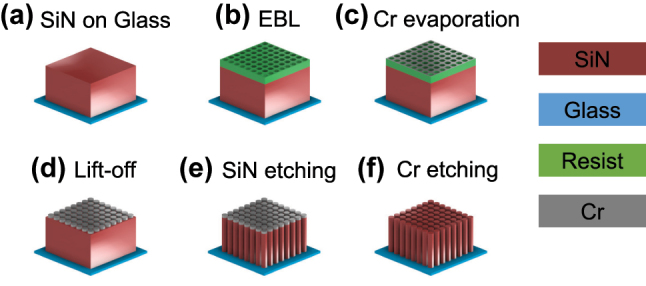
Schematic illustration of the fabrication process.

The metasurface holograms were fabricated along with the process flow shown in Figure 4. A 20-mm-square fused silica glass substrate with a thickness of 725 µm is covered with the SiN film with a thickness of 1500 nm by a sputter deposition (a). The substrate was spin-coated with 200-nm-thick positive electron beam resist (ZEP520A-7, Zeon Co., Japan) and an antistatic polymer (Espacer 300Z, Resonac Co., Japan). The hologram pattern is directly drawn using a rapid large-area electron beam lithography apparatus (F7000S-VD02, Advantest Co., Japan) with the octagonal CP (b). The pattern was transferred to a 50-nm-thick chromium film mask through vacuum evaporation (c) and lift-off process using an organic solvent, dimethylacetamide, and a stripping solution (ST-120, Tokyo Ohka Co., Japan) (d). The SiN pillars were defined using an inductively coupled plasma reactive ion etching (RIE) apparatus NE-550 (Ulvac Co., Japan) with chromium masks (e). Finally, the unnecessary chromium mask pattern was removed by a wet etchant (Cr-201, Kanto Chemical Co., Japan) for 180 s (f).

As discussed later, the sidewall verticality of the SiN nanopillars is very important to improve the diffraction efficiency of the hologram. In order to achieve this, the RIE conditions in step (e) were optimized. In particular, it is important to adjust the gas species, flow rates, bias power, and antenna power to balance the isotropic etching component that contributes to reverse taper and the anisotropic etching component that contributes to taper. We used 5 sccm of SF_6_, 25 sccm of CHF_3_, 30 W of the bias power, and 125 W of the antenna power, respectively.

## 4 Results and discussion

In this section, we first describe holograms for full-color static images that achieved high resolution, high efficiency, and high color reproducibility and compare them with the light-utilization efficiency of previous studies. Then, holograms for full-color movie reconstruction are demonstrated, and the comparison with the other multi-frame holograms is discussed in terms of the number of frames, playback speed, and resolution.

### 4.1 Reconstruction of a full-color static image

**Figure 5:**
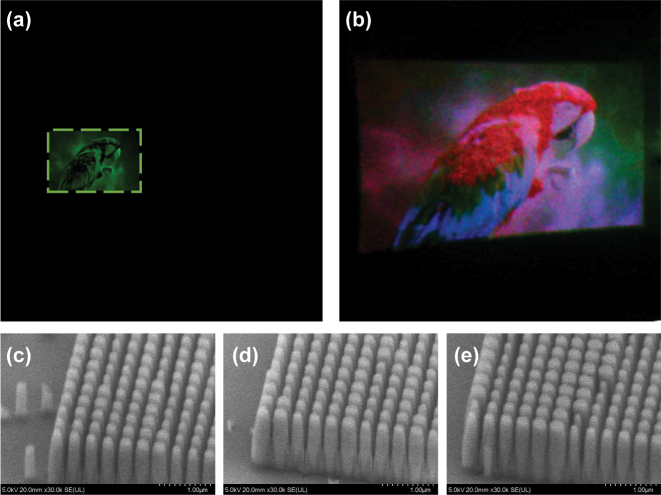
Results of full-color static image reconstruction. (a) The target image of the green channel and (b) the reconstructed image of the holograms. The scanning electron microscope images of the metasurface hologram sequences for each wavelength of (c) 445 nm, (d) 532 nm, and (e) 633 nm.

**Figure 6:**
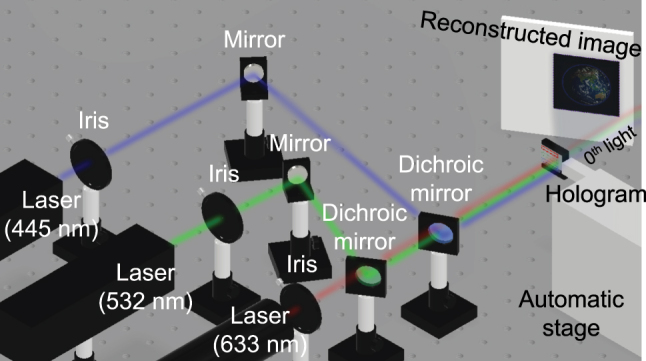
Optical setup for the full-color holographic movie reconstruction.

Figure 5(a) shows the target image of a parrot with the resolution of 5760 pixels × 5760 pixels, resized and black-padded from the original image [60]. Figure 5(b) shows the reconstructed image using an optical setup of Figure 6. Figure 5(c–e) shows the scanning electron microscope (SEM) images of the Parrot’s holograms for the wavelength channels of 445 nm (c), 532 nm (d), and 633 nm (e). As shown in this figure, optimization of the RIE conditions resulted in fabrication with good verticality.

Table 1 summarizes the transmittances and diffraction efficiencies of each color channel. The intensities of the desired +1st-order diffraction were highest for all wavelength channels. Table 2 summarizes the light utilization efficiency (transmittance × +1st diffraction efficiency) of the multicolor metasurface holograms for each color channel in comparison with previous studies. The highest efficiencies were obtained in the blue and red channels. Although lower than TiO_2_ nanoparticle embedded resin (nano-PER) in the green channel, it should be noted that this paper uses the Pancharatnam–Berry phase [27], which limits the incident circular polarization to a specific direction. If the incident polarization is not specified, the efficiency of this paper is halved, which gives an advantage to our results.

**Table 1:** Transmittance and diffraction efficiency of holograms for static images at each wavelength channel.

Wavelength	Transmittance	Diffraction efficiency [%]		
[nm]	[%]	+1st	−1st	0th
445	90.4	72.2	14.5	13.3
532	92.7	74.5	12.1	13.4
633	93.0	71.4	9.6	19.0
Average	92.0	72.7	12.1	15.2

**Table 2:** Comparison of the light utilization efficiency of multicolor metasurface holograms for each color channel.

Ref.	Materials	Efficiency [%]		
		Blue	Green	Red
[61]	TiO_2_	2.6	4.7	4.8
[62]	Si	3.6	5.2	18.0
[63]	c-Si	6.4	7.8	10.3
[64]	Si	20.0	30.0	49.0
[27]	TiO_2_ nano-PER	51.6	75.5	61.7
This work	SiN	65.3	69.1	66.4

### 4.2 Reconstruction of a full-color holographic movie

**Figure 7:**
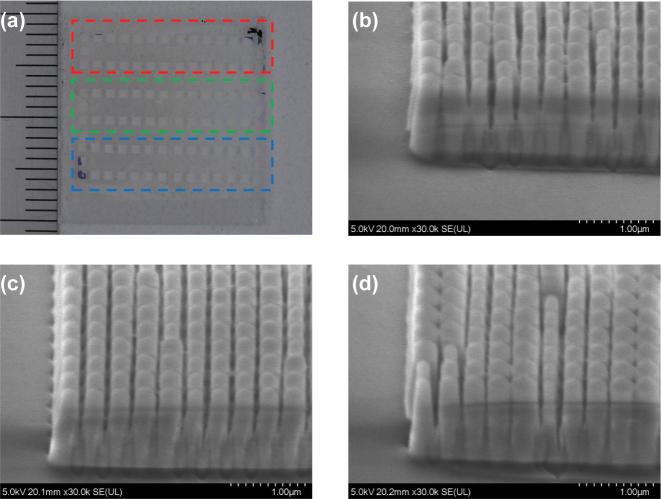
The picture and SEM images of holograms for a full-color movie. (a) The photograph after fabrication of 30 frames of metasurface holograms for each wavelength is produced on the same substrate and the SEM images of the metasurface hologram sequences for each wavelength of (b) 445 nm, (c) 532 nm, and (d) 633 nm.

Figure 7 shows the picture (a) and SEM images (b–d) of fabricated holograms for a full-color holographic movie. In this fabrication lot, the verticality was slightly worse, and the taper was more pronounced than in Figure 5.

**Figure 8:**
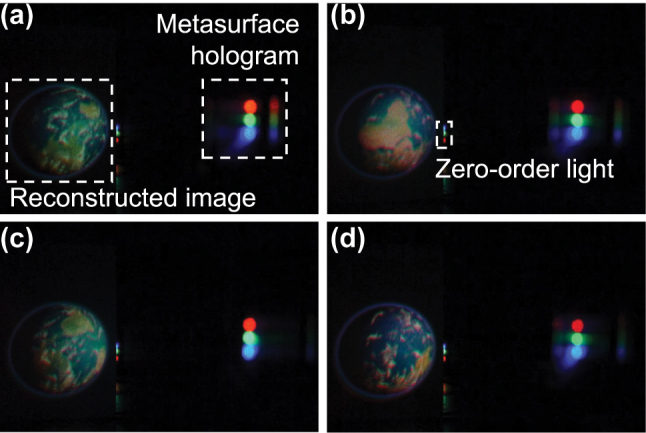
Selected reconstructed images at (a) frame 1, (b) frame 8, (c) frame 15 and (d) frame 22.

The reconstruction of a full-color movie was performed using the optical setup in Figure 6. Three-color lasers (wavelengths of 445 nm, 532 nm, and 633 nm) are irradiated on a frame on the specific channels, and an automatic stage sequentially scans the irradiation position to reconstruct the movie. Selected frames of the reconstructed movie are shown in Figure 8. The distance between the centers of adjacent frames was 0.895 mm, and the maximum speed of the automatic stage was 50 mm/s, resulting in a frame rate of 55.9 fps for the reconstructed movie. The obtained reconstructed images matched well with the target images. The movies with two frame rates are available in the Supplementary Material, Movie 1 (55.9 fps) and Movie 2 (32.9 fps). Table 3 summarizes the transmittances and diffraction efficiencies of a selected frame for each channel. The efficiency is slightly lower than in Table 1, reflecting the deterioration of verticality. Although the efficiency is slightly lower than in Table 1 reflecting the deterioration of verticality, it is higher than the other studies in Table 2. Based on these results, we have succeeded in a highly efficient full-color holographic movie using silicon nitride metasurface with a constant pitch.

**Table 3:** Transmittance and diffraction efficiency of holograms for full-color movie at each wavelength.

Wavelength	Transmittance	Diffraction efficiency [%]		
[nm]	[%]	+1st	−1st	0th
445	74.2	43.2	1.7	55.0
532	79.9	50.5	9.0	40.5
633	79.3	51.1	7.4	41.6

Table 4 summarizes the performance of this paper compared to other multi-frame metasurface holography. As shown here, the advantage of this method is high resolution to reconstruct complex image frames while maintaining sufficient playback speed and frame numbers for movie observation with the naked eye, with high color reproducibility and efficiency.

**Table 4:** Comparison of performance indicators of multi-frame metasurface holography.

Method	Reference	Wavelength [nm]	Frame numbers	Playback speed [fps]	Resolution
Expansion	[30]	633	3	0.36	Letters, shapes
Electro-optic	[34]	633	4	24.1	Letters
Chemical reaction	[37]	633	2	0.14	Shapes
Phase change materials	[39]	1550	2	–	Letters
	[40]	650	4	–	Letters
Axial spatial multiplexing	[27]	450, 532, 635	18	–	990 × 990
Polarization	[42]	532	2	–	700 × 600
Incident angle	[45]	633	4	–	Letters
Topological charge	[48]	633	60	26.5	Shapes
In-plane spatial multiplexing	[49]	633	28	9523	400 × 400
	[50]	633	48	30	2048 × 2048
	[51]	445, 532, 633	20	30	1920 × 1080
	This work	445, 532, 633	30	55.9	2322 × 2322

## 5 Conclusions

In this study, we achieved the reconstruction of a full-color dielectric metasurface holographic movie with a resolution of 2322 × 2322 by spatiotemporally multiplexing 30 frames, utilizing blue, green, and red color channels at wavelengths of 445 nm, 532 nm, and 633 nm, respectively. The superposition of three wavelengths was accomplished by adjusting the resolutions and positions of target images for each wavelength while keeping the meta-atom pitch constant. By utilizing polarization-independent silicon nitride waveguide meta-atoms, we were able to obtain a high average transmittance and diffraction efficiency, measuring at 92.0 % and 72.7 %, respectively. The results are expected to contribute to the future projection of three-dimensional metasurface holographic movie.
